# Medical cannabis use in the United States: a retrospective database study

**DOI:** 10.1186/s42238-020-00038-w

**Published:** 2020-09-29

**Authors:** V. Kishan Mahabir, Jamil J. Merchant, Christopher Smith, Alisha Garibaldi

**Affiliations:** CB2 Insights, 5045 Orbitor Dr, Building 11, Suite 300, Mississauga, ON L4W 4Y4 Canada

**Keywords:** Medical cannabis, Chronic pain, Anxiety, Post-traumatic stress disorder, Gender

## Abstract

**Introduction:**

Growing interest in the medicinal properties of cannabis has led to an increase in its use to treat medical conditions, and the establishment of state-specific medical cannabis programs. Despite medical cannabis being legal in 33 states and the District of Colombia, there remains a paucity of data characterizing the patients accessing medical cannabis programs.

**Methods:**

We retrospectively reviewed a registry with data from 33 medical cannabis evaluation clinics in the United States, owned and operated by CB2 Insights. Data were collected primarily by face-to-face interviews for patients seeking medical cannabis certification between November 18, 2018 and March 18, 2020. Patients were removed from the analysis if they did not have a valid date of birth, were less than 18, or did not have a primary medical condition reported; a total of 61,379 patients were included in the analysis. Data were summarized using descriptive statistics expressed as a mean (standard deviation (SD)) or median (interquartile range (IQR)) as appropriate for continuous variables, and number (percent) for categorical variables. Statistical tests performed across groups included t-tests, chi-squared tests and regression.

**Results:**

The average age of patients was 45.5, 54.8% were male and the majority were Caucasian (87.5%). Female patients were significantly older than males (47.0 compared to 44.6). Most patients reported cannabis experience prior to seeking medical certification (66.9%). The top three mutually exclusive primary medical conditions reported were unspecified chronic pain (38.8%), anxiety (13.5%) and post-traumatic stress disorder (PTSD) (8.4%). The average number of comorbid conditions reported was 2.7, of which anxiety was the most common (28.3%). Females reported significantly more comorbid conditions than males (3.1 compared to 2.3).

**Conclusion:**

This retrospective study highlighted the range and number of conditions for which patients in the US seek medical cannabis. Rigorous clinical trials investigating the use of medical cannabis to treat pain conditions, anxiety, insomnia, depression and PTSD would benefit a large number of patients, many of whom use medical cannabis to treat multiple conditions.

## Background

The cannabis plant has been used in traditional medicine for centuries, and within the last few decades it has generated considerable attention among the general population, modern medical community and regulatory bodies for its potential medicinal capabilities (Alsherbiny and Li 2018). The effects of cannabis are due to the action of cannabinoids, a diverse group of chemical compounds found in the cannabis plant that act on the human endocannabinoid system, via a series of interactions with cell receptors throughout the human body, and alter neurotransmitter release in the brain affecting various physiological functions (Fraguas-Sanchez and Torres-Suarez 2018; Vuckovic et al. 2018). While more than 100 cannabinoids have been identified, Δ-9-tetrahydrocannabinol (THC) and cannabidiol (CBD) have undergone the most scientific investigation and are considered to be the greatest contributors to the medicinal effects of cannabis (Pertwee et al. 2010; Pertwee 1997).

The growing interest in medical cannabis has led to an increase in its use to treat medical conditions or symptoms thereof, such as chronic pain, anxiety, and depression. Individuals do this either through self-medication, accessing the drug via the recreational or illicit markets, or via medical cannabis programs in regions where regulations permit. Other clinical conditions that cannabis is thought to treat include multiple sclerosis, AIDS-associated wasting/cachexia, insomnia, arthritis, epilepsy, post-traumatic stress disorder (PTSD), glaucoma, headaches and migraines, and nausea (Kosiba et al. 2019; Lu and Anderson 2017; Kaur et al. 2016; Zaller et al. 2015; Klumpers and Thacker 2019; Institute of Medicine (U.S.) 1999).

Medical cannabis programs began in the United States (US) in 1996, with California becoming the first state to legalize medical cannabis (Legislatures NC of S 2020). Since then, other states have slowly adopted medical cannabis programs, with the programs themselves evolving over time. As of 2000, 8 states had legalized medical cannabis (Yu et al. 2020); by 2010, there were 16 and by 2016, there were 29 (Pacula and Smart 2017). As of July 2020, medical cannabis is legal in 33 states and the District of Columbia, 12 of which allow adults over the age of 21 to use cannabis recreationally (DISA Global Solutions 2019).

Qualifying conditions for medical cannabis vary significantly state-by-state as some states (e.g., California, Massachusetts, Oklahoma, and the District of Columbia) allow physicians to use discretion when recommending patients for certification, while other states only allow certification based on a limited set of qualifying conditions (Legislatures NC of S 2020). The allowable THC-percentage component of state-run programs also varies, with certain states only allowing access to high-CBD, low-THC products for medical cannabis patients. Patients seeking medical cannabis in the US in most states are required to obtain a state-specific medical cannabis identification card, allowing them to purchase cannabis products from dispensaries to treat certain medical conditions.

Despite medical cannabis being legal in many states, there remains a paucity of data characterizing the patients accessing it via state-run programs. Two large studies reviewed available state registry data of patients holding medical cannabis licenses; however, these studies came with limitations including voluntary reporting at the state-level, or inability for the authors to access the registry data (Boehnke et al. 2019; Fairman 2016). One of the studies reviewed the primary conditions for which patients sought medical cannabis, but did not report any other patient characteristics such as age or gender (Boehnke et al. 2019). The other reported on the age and gender of patients accessing medical cannabis, but did not report on medical conditions (Fairman 2016). While useful, these studies did not adequately characterize medical cannabis use through state programs by age, gender or condition. Other studies that have been published to characterize medical cannabis patients are limited by sample size and selection, include only patient-reported data, or include patients outside of the US (Sexton et al. 2016; Eurich et al. 2019; Bonn-Miller et al. 2014; Reinarman et al. 2011).

Given the need for a large data set to contribute to the medical knowledge, inform on policy and identify areas for future research, we designed a retrospective study of a registry database. The primary objective of this study was to thoroughly describe the population of patients seeking treatment with medical cannabis in the US. These data were reviewed at a high-level to answer the following questions:

1. What are the key demographic characteristics of patients accessing medical cannabis?
2. Are there differences in characteristics of males and females accessing medical cannabis?
3. What are the most commonly reported conditions among this sample of patients?
4. How many conditions do patients seek treatment for, and does this change based on age and gender?

These questions were investigated to assist the medical community in further developing an understanding of patients seeking medical cannabis for treatment of their conditions and symptoms, and to assist others in determining areas of interest for future research. This knowledge may also inform policy makers in states considering medical cannabis legalization or the further development of existing medical cannabis programs in states where medical cannabis has already been legalized.

## Methods

This was a retrospective database study of patients seeking medical cannabis certification in the US. Data were extracted from the database software utilized in CB2 Insights’ clinical network. CB2 Insights operates one of the largest collections of medical cannabis evaluation clinics in the US, collectively assessing approximately 100,000 patients per year seeking access to medical cannabis, using a single and consistent software that contributes data to a patient registry. These 33 independent clinics are not connected to dispensaries or producers of medical cannabis, and are situated across 12 states (number of clinics): Colorado (6), Connecticut (1), Delaware (2), Illinois (1), Maine (1), Maryland (1), Massachusetts (10), Missouri (1), New Jersey (5), New York (1), Rhode Island (2), and Pennsylvania (2). Patients access these clinics by physician-referral or self-referral through word of mouth, community out-reach and marketing. Over 95% of data were collected via face-to-face interview, with the remaining collected via telemedicine. Patients presenting to any of the clinics are required to complete the same baseline information upon intake, including demographic, medical, and therapeutic information; however, certain characteristics such as race and gender were not made mandatory initially, and are not reported for all patients. Baseline questions include patient-reported tobacco smoking and alcohol use, current or past substance abuse of drugs and/or alcohol, use of illicit (illegal) drugs, medication use and alternative therapies. Medication use is an open-ended question that may be completed by transcribing a medication list into the software, which leaves room for errors and may be a limitation of the data. All patients indicate their primary reason for seeking access to medical cannabis and are asked to report all comorbid conditions for which they are also seeking medical cannabis. Patients are required to provide supporting documentation of their medical histories and relevant conditions for review and verification, in the form of medical records or a letter from another physician. Review of medical documentation, in combination with a medical evaluation by a state-authorized physician or nurse practitioner are used to confirm their qualification for medical cannabis within their respective state. Prior to data export, the protocol was reviewed by the Advarra Institutional Review Board (IRB) and was determined to be exempt from IRB oversight (**Pro00042652**) as the study had minimal risk, the data exports were void of patient identifiers, and it did not require direct patient contact.

**Fig. 1:**
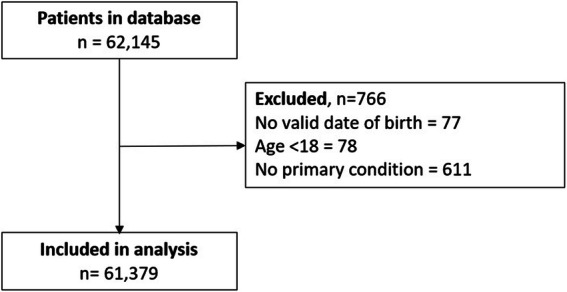
Patient Flow

Data were exported for 62,145 patients who were seen for their initial assessment between November 18, 2018 (when the technology and standardized protocol were introduced into the clinics) and March 18, 2020. Data were exported without any patient identifiers to ensure patient anonymity. Eligibility criteria were applied to the data set and the following patients were removed: 1) 77 patients without a valid date of birth; 2) 78 patients younger than 18; and 3) 611 patients without a primary medical condition reported. Overall, 61,379 patients were included in the analysis (Fig. 1).

Data from the database software utilized in CB2 Insights’ clinical network were also merged with US tax data, which provides tabulations of income tax data by ZIP code in order to estimate household income based on individual patients’ ZIP codes. Median household income values from the 2018 dataset purchased from Cubit Planning Inc. were used (US Income Statistics - Current Census Data for Zip Codes 2018). Cubit Planning Inc. summarizes the most current income statistics from the US Census Bureau.

When a final dataset was confirmed, data were analyzed using RStudio (Boston, MA). All information was summarized using descriptive statistics expressed as a mean (standard deviation (SD)) or median (interquartile range (IQR)) as appropriate for continuous variables, and number (percent) for categorical variables. Univariate analyses were conducted to inform multivariate analyses including t-tests when comparing means and chi-squared tests when comparing proportions. Regression analyses were conducted to determine if age and gender, specifically, were significant predictors of characteristics of smoking, alcohol consumption, prior cannabis use and medication usage, the number of medications being used, and the number of conditions reported. Logistic regression was used for dichotomous variables and linear regression was used for continuous variables. To analyze whether age and gender were significant predictors of reporting each primary condition, each condition was compared separately to all others using logistic regression. An interaction model with age and gender was included for all regression analyses; if the interaction effect was significant, *p*-values are reported for the interaction model, otherwise p-values are reported for the model without interaction. All tests were completed with a significance level of 0.05. *P*-values less than 0.001 are expressed as *p* < 0.001, and 95% confidence intervals (CI) are provided where appropriate.

## Results

The average age of patients in the sample was 45.5 (SD = 15.8) and 54.8% were male (Table 1). The average age of females, 47.0 (SD = 15.7), was significantly greater than males, 44.6 (SD = 15.7) (*p* < 0.001, difference in means = 2.4, 95% CI: 2.15–2.68) (Table 2). Of the patients with race reported, Caucasians represented the largest group of the sample population at 87.5%. The median household income in the ZIP code in which patients resided was available for 56,083 patients. The overwhelming majority of patients lived in a ZIP code where the median household income was above $40,000 (93.7%); the median was $69,481 (IQR $35,807). Most patients (66.9%) reported that they had experience with cannabis prior to seeking medical certification, were non-smokers (81.2%), did not drink (57.5%) and did not have a history of substance abuse (94.4%). Gender was not a significant predictor of reporting prior cannabis experience or history of substance abuse (*p* = 0.929 and 0.871, respectively), but was for smoking status and alcohol consumption (*p* < 0.001) (Tables 3 and 4). Males reported smoking tobacco more than females, whereas females reported the use of alcohol more than males.

**Table 1:** Sociodemographic and medical characteristics of 61,379 patients seeking medical cannabis certification from CB2 Insights evaluation clinics

Characteristic	Patients (***n*** = 61,379)
	Mean (SD) or n (%)
Age (years), mean (SD)	45.5 (15.8)
Gender	
Male	33,651 (54.8%)
Female	23,209 (37.8%)
Non-binary	33 (0.1%)
Unknown	4486 (7.3%)
Race (*n* = 32,275)	
White/Caucasian	28,322 (87.5%)
Black/African American	2738 (8.5%)
Other	518 (1.6%)
Asian	277 (0.9%)
American Indian/Alaska Native	261 (0.8%)
Middle Eastern	63 (0.2%)
Native Hawaiian or Other Pacific Islander	58 (0.2%)
South East Asian	38 (0.1%)
Surrogate Household Income (*n* = 56,083)	
< $20,000	14 (0.0%)
$20,000- < $40,000	3519 (6.3%)
$40,000- < $60,000	15,660 (27.9%)
$60,000- < $80,000	17,318 (30.9%)
$$80,000- < $100,000	10,205 (18.2%)
> $100,000	9367 (16.7%)
Smoking Status	
Smoker	11,509 (18.8%)
Non-smoker	49,870 (81.2%)
Alcohol Consumption	
Yes	26,081 (42.5%)
No	35,298 (57.5%)
Previous Cannabis Experience	
Yes	41,070 (66.9%)
No	20,309 (33.1%)
Use of Non-Cannabis Illicit Drugs	
Yes	274 (0.4%)
No	61,105 (99.6%)
History of Substance Abuse	
Yes	3426 (5.6%)
No	57,943 (94.4%)
Number of Medications	
0	34,273 (55.8%)
1	7866 (12.8%)
2	4755 (7.7%)
3	3515 (5.7%)
4	2372 (3.9%)
5+	8598 (14.0%)
Alternate Therapies	
Exercise	25,831 (42.1%)
Massage therapy	13,250 (21.6%)
Mental health counselling	11,235 (18.3%)
Chiropractor	11,047 (18.0%)
Acupuncture	5632 (9.2%)
Mindfulness-based cognitive therapy	5176 (8.4%)
Aroma therapy	3849 (6.3%)
Cognitive behavioural therapy	3470 (5.7%)
Physiotherapy	3380 (5.5%)
Homeopathic medicine	3186 (5.2%)
Reiki	2329 (3.8%)
Naturopathic medicine	1788 (2.9%)
Addictions counselling	1158 (1.9%)
Other	221 (0.4%)
None	24,939 (40.6%)
Number of Comorbid Conditions	
0	10,807 (17.6%)
1	15,971 (26.0%)
2	9863 (16.1%)
3	7319 (11.9%)
4	5497 (9.0%)
5+	11,922 (19.4%)

Table 1 summarizes key patient characteristics of the entire sample of 61,379 patients who received medical cannabis certification at clinics owned and operated by CB2 Insights. For characteristics in which data were not available for every patient, a sample size for that variable is provided. SD = standard deviation

**Table 2:** Univariate analysis of differences of key characteristics of 61,379 patients seeking medical cannabis certification by age

Characteristic	Average Age (SD)	p-value, 95% CI around the difference in means
Gender		
Male	44.6 (15.7)	< 0.001 2.15–2.68
Female	47.0 (15.7)	
Smoking Status		
Smoker	42.6 (13.8)	< 0.001 3.28–3.86
Non-smoker	46.2 (16.1)	
Alcohol Consumption		
Yes	45.7 (15.6)	0.004 0.06–0.12
No	45.3 (15.9)	
Previous Cannabis Experience		
Yes	45.1 (15.5)	< 0.001 0.80–1.42
No	46.3 (16.4)	
History of Substance Abuse		
Yes	42.5 (13.7)	< 0.001 2.70–3.65
No	45.7 (15.9)	
Medication Usage		
None	42.9 (15.5)	< 0.001 6.15–5.66
At least one	48.8 (15.6)	

Table 2 summarizes the analysis of differences of key patient characteristics by age. T-tests were conducted for differences in average age for all characteristics. *SD* standard deviation, *CI* confidence interval. CIs are provided around the differences in the means

**Table 3:** Univariate analysis of differences of key characteristics of 56,860 patients seeking medical cannabis certification by gender

Characteristic	Male ***n*** = 33,651	Female ***n*** = 23,209	p-value
	n (%) or mean (SD)	n (%) or mean (SD)	
Smoking Status			
Smoker	6619 (19.7%)	4063 (17.5%)	< 0.001
Non-smoker	27,032 (80.3%)	19,146 (82.5%)	
Alcohol Consumption			
Yes	13,880 (41.2%)	9971 (43.0%)	< 0.001
No	19,771 (58.8%)	13,238 (57.0%)	
Previous Cannabis Experience			
Yes	23,553 (70.0%)	15,474 (66.7%)	< 0.001
No	10,098 (30.0%)	7735 (33.3%)	
History of Substance Abuse			
Yes	2092 (6.2%)	997 (4.3%)	< 0.001
No	31,559 (93.8%)	22,212 (95.7%)	
Medication Usage			
None	21,268 (63.2%)	11,010 (47.4%)	< 0.001
At least one	12,383 (36.8%)	12,199 (52.6%)	
Average Number of Medications* (*n* = 27,106)	4.0 (3.6)	4.4 (3.7)	< 0.001
Average Number of Conditions	3.3 (2.3)	4.1 (2.9)	< 0.001

Table 3 summarizes the univariate analysis of differences of key patient characteristics by gender among 56,860 patients for whom gender was reported. Percentages are calculated from the sample size for each respective column. Chi-squared tests were conducted for differences in proportions of males and females for all characteristics, except average number of medications and average number of conditions, in which t-tests were conducted. SD = standard deviation. *Average number of medications is calculated for those reporting at least one medication (*n* = 27,106)

**Table 4:** Multivariate analysis results of differences of key characteristics of 56,860 patients seeking medical cannabis certification by gender and age, with and without interaction

	Model without interaction between age and gender		Model with interaction between age and gender	
Characteristic	Coefficient (95% CI)	p-value	Coefficient (95% CI)	p-value
Smoking Status				
Intercept	−0.890 (− 0.961, − 0.820)	< 0.001	−1.00 (−1.108, − 0.900)	< 0.001
Age	− 0.014 (− 0.016, − 0.013)	< 0.001	− 0.012 (− 0.014, − 0.010)	< 0.001
Gender (male)	0.110 (0.066, 0.153)	< 0.001	0.294 (0.163, 0.426)	< 0.001
Interaction	NA	NA	− 0.004 (− 0.007, − 0.001)	0.004
Alcohol Consumption				
Intercept	−0.368 (− 0.424, − 0.311)	< 0.001	−0.220 (− 0.302, − 0.138)	< 0.001
Age	0.002 (0.001, 0.003)	< 0.001	− 0.001 (− 0.003, 0.000)	0.108
Gender (male)	− 0.066 (− 0.100, − 0.032)	< 0.001	−0.312 (− 0.417, − 0.207)	< 0.001
Interaction	NA	NA	0.005 (0.003, 0.007)	< 0.001
Previous Cannabis Experience				
Intercept	0.904 (0.844, 0.964)	< 0.001	0.992 (0.905, 1.08)	< 0.001
Age	−0.004 (− 0.006, − 0.003)	< 0.001	− 0.006 (− 0.008, − 0.005)	< 0.001
Gender (male)	0.143 (0.107, 0.179)	< 0.001	− 0.005 (− 0.117, 0.107)	0.929
Interaction	NA	NA	0.003 (0.001, 0.005)	0.006
History of Substance Abuse				
Intercept	−2.540 (−2.664, −2.418)	< 0.001	−2.284 (−2.475, −2.095)	< 0.001
Age	−0.012 (− 0.015, − 0.010)	< 0.001	−0.018 (− 0.022, − 0.014)	< 0.001
Gender (male)	0.361 (0.284, 0.439)	< 0.001	−0.019 (− 0.249, 0.212)	0.871
Interaction	NA	NA	0.009 (0.004, 0.014)	0.001
Medication Usage				
Intercept	−1.014 (− 1.072, − 0.957)	< 0.001	−0.595 (− 0.677, − 0.513)	< 0.001
Age	0.024 (0.023, 0.025)	< 0.001	0.015 (0.013, 0.017)	< 0.001
Gender (male)	−0.607 (− 0.642, − 0.573)	< 0.001	− 1.336 (− 1.445, − 1.227)	< 0.001
Interaction	NA	NA	0.016 (0.013, 0.018)	< 0.001
Average Number of Medications				
Intercept	0.236 (0.214, 0.259)	< 0.001	0.356 (0.324, 0.389)	< 0.001
Age	0.011 (0.011, 0.012)	< 0.001	0.009 (0.008, 0.009)	< 0.001
Gender (male)	−0.233 (− 0.247, − 0.220)	< 0.001	−0.432 (− 0.473, − 0.391)	< 0.001
Interaction	NA	NA	0.004 (0.003, 0.005)	< 0.001
Average Number of Conditions				
Intercept	1.483 (1.469, 1.497)	< 0.001	1.507 (1.487, 1.527)	< 0.001
Age	0.000 (0.000, 0.000)	0.423	−0.001 (− 0.001, 0.000)	0.003
Gender (male)	−0.143 (− 0.151, − 0.135)	< 0.001	−0.183 (− 0.209, − 0.157)	< 0.001
Interaction	NA	NA	0.001 (0.000, 0.001)	0.001

Table 4 shows the results of regression analysis for age and gender as predictors for variables analyzed with univariate analysis in Tables 2 and 3 among 56,860 patients for whom gender was reported. Results are presented with and without an interaction between age and gender included in the model. The coefficient column represents the magnitude of effect and direction of the predictor variable; a negative coefficient for age suggests that younger patients are more likely to report the characteristic, and a negative coefficient for gender suggests that females are more likely to report the characteristic. *CI* confidence interval

Less than half of patients reported prescription medication use (44.2%). Increased age and female gender were significant predictors of reporting at least one medication (*p* < 0.001) and a greater number of medications (p < 0.001) (Tables 3 and 4). Of patients who reported taking at least one medication (*n* = 27,106), the mean number of medications reported was 4.1 (SD = 3.7). Over half the sample (59.4%) reported currently using an alternate form of therapy. Of those who reported using a therapy, the average number reported was 2.5 (SD = 1.7), and the most commonly reported among this group were exercise (70.9%), massage therapy (36.4%), mental health counselling (30.8%) and chiropractor (30.3%). A quarter of the sample (26.1%) did not report any current use of medications or alternate therapy.

Regardless of gender, the top three primary medical conditions were unspecified chronic pain (*n* = 23,817, 38.8%), anxiety (*n* = 8280, 13.5%) and PTSD (*n* = 5143, 8.4%) (Table 5). Following the top three were back and neck problems (*n* = 3969, 6.5%), arthritis (*n* = 2395, 3.9%), insomnia (*n* = 2096, 3.4%) and cancer-related pain (*n* = 1641, 2.7%). Depression, migraines, muscle spasms, ADD/ADHD, chronic nausea, fibromyalgia, headaches and epilepsy were each reported as the primary medical condition for 2.0% or less of the sample. Of the primary medical conditions, 10.6% of those reported were other medical conditions each representing less than 1.0% of the entire sample. Gender was not a significant predictor of epilepsy, but was a significant predictor for all other conditions (Table 6); females were significantly more likely to report anxiety, PTSD, arthritis, cancer related pain, depression, migraines, chronic nausea, fibromyalgia and headaches, whereas males were significantly more likely to report unspecified chronic pain, back & neck problems, insomnia, muscle spasms and ADD/ADHD.

**Table 5:** Primary medical condition reported by 61,379 patients seeking medical cannabis certification, by gender

Primary Condition	Patients, n (%)	Male, n (%)	Female, n (%)
	***n*** = 61,379	***n*** = 33,651	***n*** = 23,209
Unspecified Chronic Pain	23,817 (38.8%)	14,164 (42.1%)	8710 (37.5%)
Anxiety	8280 (13.5%)	3949 (11.7%)	3224 (13.9%)
Post Traumatic Stress Disorder	5143 (8.4%)	2874 (8.5%)	1982 (8.5%)
Back & Neck Problems	3969 (6.5%)	2506 (7.4%)	1125 (4.8%)
Arthritis	2395 (3.9%)	1149 (3.4%)	1048 (4.5%)
Insomnia	2096 (3.4%)	1187 (3.5%)	673 (2.9%)
Cancer Related Pain	1641 (2.7%)	782 (2.3%)	722 (3.1%)
Depression	1249 (2.0%)	575 (1.7%)	470 (2.0%)
Migraines	1245 (2.0%)	499 (1.5%)	656 (2.8%)
Muscle Spasms	1038 (1.7%)	624 (1.9%)	384 (1.7%)
ADD/ADHD	1002 (1.6%)	624 (1.9%)	235 (1.0%)
Chronic Nausea	926 (1.5%)	477 (1.4%)	419 (1.8%)
Fibromyalgia	726 (1.2%)	70 (0.2%)	597 (2.6%)
Headaches	707 (1.2%)	356 (1.1%)	307 (1.3%)
Epilepsy	626 (1.0%)	372 (1.1%)	224 (1.0%)
Other	6519 (10.6%)	3443 (10.2%)	2433 (10.5%)

Table 5 presents a summary of the individuals reporting each primary condition overall and by gender. Patients could only report one primary condition. Any condition representing less than 1.0% was grouped as an “other”. A list of “other” primary conditions is available in Table 8. Percentages are calculated from the sample size for each respective column. *ADD* attention deficit disorder, *ADHD* attention deficit hyperactivity disorder

**Table 6:** Multivariate analysis results of differences in primary condition reported by 56,860 patients seeking medical cannabis certification by gender and age, with and without interaction

Primary Condition	Model without interaction between age and gender		Model with interaction between age and gender	
	Coefficient (95% CI)	p-value	Coefficient (95% CI)	p-value
Unspecified Chronic Pain				
Intercept	− 0.500 (− 0.557, − 0.443)	< 0.001	− 0.647 (− 0.731, − 0.563)	< 0.001
Age	0.000 (− 0.001, 0.001)	0.700	0.003 (0.001, 0.005)	0.001
Gender (Male)	0.190 (0.156, 0.224)	< 0.001	0.430 (0.323, 0.536)	< 0.001
Interaction	NA	NA	− 0.005 (− 0.007, − 0.003)	< 0.001
Anxiety				
Intercept	− 1.047 (− 1.128, − 0.966)	< 0.001	− 0.972 (− 1.085, − 0.859)	< 0.001
Age	−0.017 (− 0.019, − 0.015)	< 0.001	−0.019 (− 0.021, − 0.016)	< 0.001
Gender (Male)	−0.236 (− 0.286, − 0.185)	< 0.001	−0.369 (− 0.519, − 0.220)	< 0.001
Interaction	NA	NA	0.003 (0.000, 0.006)	0.064
Post Traumatic Stress Disorder				
Intercept	−1.345 (− 1.442, − 1.249)	< 0.001	−0.873 (− 1.011, − 0.737)	< 0.001
Age	− 0.023 (− 0.025, − 0.021)	< 0.001	−0.034 (− 0.038, − 0.031)	< 0.001
Gender (Male)	−0.054 (− 0.114, 0.006)	0.079	−0.846 (− 1.024, − 0.669)	< 0.001
Interaction	NA	NA	0.019 (0.015, 0.023)	< 0.001
Back & Neck Problems				
Intercept	−3.718 (− 3.841, − 3.596)	< 0.001	−3.772 (− 3.977, − 3.571)	< 0.001
Age	0.015 (0.013, 0.017)	< 0.001	0.016 (0.012, 0.020)	< 0.001
Gender (Male)	0.495 (0.423, 0.568)	< 0.001	0.572 (0.333, 0.814)	< 0.001
Interaction	NA	NA	−0.002 (− 0.006, 0.003)	0.508
Arthritis				
Intercept	−6.013 (− 6.201, − 5.828)	< 0.001	−6.081 (− 6.355, − 5.813)	< 0.001
Age	0.056 (0.053, 0.059)	< 0.001	0.057 (0.052, 0.061)	< 0.001
Gender (Male)	−0.179 (− 0.266, − 0.092)	< 0.001	−0.057 (− 0.417, 0.304)	0.755
Interaction	NA	NA	−0.002 (− 0.008, 0.004)	0.493
Insomnia				
Intercept	−3.980 (− 4.143, − 3.818)	< 0.001	− 4.620 (− 4.892, − 4.355)	< 0.001
Age	0.010 (0.007, 0.013)	< 0.001	0.022 (0.017, 0.027)	< 0.001
Gender (Male)	0.226 (0.130, 0.323)	< 0.001	1.190 (0.871, 1.513)	< 0.001
Interaction	NA	NA	−0.020 (− 0.026, − 0.013)	< 0.001
Cancer Related Pain				
Intercept	−6.200 (− 6.421, − 5.983)	< 0.001	− 5.679 (− 5.978, − 5.388)	< 0.001
Age	0.052 (0.049, 0.056)	< 0.001	0.043 (0.038, 0.048)	< 0.001
Gender (Male)	−0.192 (− 0.296, − 0.088)	< 0.001	− 1.184 (− 1.604, − 0.764)	< 0.001
Interaction	NA	NA	0.017 (0.010, 0.024)	< 0.001
Depression				
Intercept	−3.026 (− 3.225, − 2.830)	< 0.001	−3.007 (− 3.282, − 2.738)	< 0.001
Age	−0.019 (− 0.023, − 0.015)	< 0.001	−0.019 (− 0.026, − 0.013)	< 0.001
Gender (Male)	−0.218 (− 0.341, − 0.094)	0.001	−0.253 (− 0.616, 0.111)	0.173
Interaction	NA	NA	0.001 (−0.007, 0.009	0.842
Migraines				
Intercept	−2.621 (− 2.805, − 2.438)	< 0.001	− 2.689 (− 2.923, − 2.459)	< 0.001
Age	−0.021 (− 0.024, − 0.017)	< 0.001	−0.019 (− 0.024, − 0.014)	< 0.001
Gender (Male)	−0.708 (− 0.827, − 0.590)	< 0.001	−0.549 (− 0.896, − 0.202)	0.002
Interaction	NA	NA	−0.004 (− 0.012, 0.004)	0.339
Muscle Spasms				
Intercept	−4.027 (− 4.240, − 3.817)	< 0.001	−4.320 (− 4.649, − 4.000)	< 0.001
Age	− 0.001 (− 0.005, 0.003)	0.542	0.005 (− 0.001, 0.011)	0.131
Gender (Male)	0.113 (− 0.015, 0.242)	0.085	0.577 (0.178, 0.981)	0.005
Interaction	NA	NA	−0.010 (− 0.018, − 0.002)	0.016
ADD/ADHD				
Intercept	− 3.030 (− 3.262, − 2.800)	< 0.001	− 3.607 (− 3.991, − 3.231)	< 0.001
Age	−0.036 (− 0.041, − 0.031)	< 0.001	−0.022 (− 0.031, − 0.014)	< 0.001
Gender (Male)	0.535 (0.385, 0.688)	< 0.001	1.359 (0.915, 1.809)	< 0.001
Interaction	NA	NA	−0.021 (− 0.032, − 0.011)	< 0.001
Chronic Nausea				
Intercept	−2.193 (− 2.404, − 1.983)	< 0.001	−2.076 (− 2.359, − 1.795)	< 0.001
Age	− 0.043 (− 0.048, − 0.038)	< 0.001	−0.046 (− 0.053, − 0.039)	< 0.001
Gender (Male)	− 0.341 (− 0.473, − 0.207)	< 0.001	− 0.567 (− 0.955, − 0.178)	0.004
Interaction	NA	NA	0.006 (− 0.004, 0.016)	0.224
Fibromyalgia				
Intercept	−4.434 (− 4.699, − 4.176)	< 0.001	−4.392 (− 4.671, − 4.121)	< 0.001
Age	0.016 (0.011, 0.021)	< 0.001	0.016 (0.010, 0.021)	< 0.001
Gender (Male)	− 2.502 (− 2.758, − 2.261)	< 0.001	− 2.868 (− 3.715, − 2.069)	< 0.001
Interaction	NA	NA	0.007 (−0.008, 0.023)	0.352
Headaches				
Intercept	−3.439 (− 3.686, − 3.195)	< 0.001	−3.632 (− 3.974, − 3.298)	< 0.001
Age	− 0.020 (− 0.025, − 0.014)	< 0.001	−0.015 (− 0.022, − 0.008)	< 0.001
Gender (Male)	−0.272 (− 0.426, − 0.118)	0.001	0.091 (− 0.363, 0.548)	0.695
Interaction	NA	NA	−0.009 (− 0.019, 0.002)	0.096
Epilepsy				
Intercept	−3.622 (− 3.889, − 3.359)	< 0.001	−3.720 (− 4.115, − 3.334)	< 0.001
Age	−0.023 (− 0.028, − 0.017)	< 0.001	−0.020 (− 0.029, − 0.012)	< 0.001
Gender (Male)	0.084 (− 0.082, 0.253)	0.323	0.242 (− 0.247, 0.736)	0.334
Interaction	NA	NA	−0.004 (− 0.015, 0.007)	0.502

Table 6 shows the results of logistic regression analysis for age and gender as predictors for primary conditions for 56,860 patients. Logistic regression was conducted for each primary condition compared to all other conditions to determine whether age and gender predict reporting the primary condition versus not reporting the primary condition. The coefficient column represents the magnitude of effect and direction of the predictor variable; a negative coefficient for age suggests that younger patients are more likely to report the primary condition, and a negative coefficient for gender suggests that females are more likely to report the primary condition. *CI* = confidence interval

Patients reporting anxiety, PTSD, depression, migraines, ADD/ADHD, chronic nausea, headaches or epilepsy as their primary reason for seeking medical cannabis were significantly more likely to be younger (*p* < 0.001), whereas patients seeking medical cannabis primarily for unspecified chronic pain, back and neck problems, arthritis, insomnia, cancer related pain or fibromyalgia were significantly more likely to be older (Table 6). Age was not a significant predictor of reporting muscle spasms.

Patients were able to report any number of comorbid medical conditions necessary to describe their reason(s) for seeking medical cannabis (Table 7). The average number of comorbid medical conditions reported was 2.7 (SD = 2.6). Anxiety was the most commonly reported comorbid condition (*n* = 17,359, 28.3%), followed by back and neck problems (*n* = 14,550, 23.7%), insomnia (n = 14,247, 23.2%), depression (*n* = 13,413, 21.9%), and unspecified chronic pain (*n* = 11,199, 18.2%). Only 17.6% of the sample did not report a comorbid medical condition.

**Table 7:** Summary of comorbid conditions and all conditions reported by 61,379 patients seeking medical cannabis certification, by gender

Condition	Patients, n (%)	Male, n (%)	Female, n (%)
	***n*** = 61,379	***n*** = 33,651	***n*** = 23,209
Comorbid Conditions			
Anxiety	17,359 (28.3%)	8306 (24.7%)	7465 (32.2%)
Back & Neck Problems	14,550 (23.7%)	7539 (22.4%)	5658 (24.4%)
Insomnia	14,247 (23.2%)	7108 (21.1%)	5689 (24.5%)
Depression	13,413 (21.9%)	5859 (17.4%)	6130 (26.4%)
Unspecified Chronic Pain	11,199 (18.2%)	5756 (17.1%)	4523 (19.5%)
Headaches	8688 (14.2%)	3889 (11.6%)	4029 (17.4%)
Arthritis	8600 (14.0%)	4105 (12.2%)	3793 (16.3%)
Muscle Spasms	7832 (12.8%)	409 (1.2%)	3257 (14.0%)
Post Traumatic Stress Disorder	6155 (10.0%)	2764 (8.2%)	2876 (12.4%)
Migraines	6063 (9.9%)	2273 (6.8%)	3276 (14.1%)
ADD/ADHD	4612 (7.5%)	2413 (7.2%)	1612 (6.9%)
Chronic Nausea	4440 (7.2%)	1892 (5.6%)	2268 (9.8%)
Fibromyalgia	1809 (2.9%)	277 (0.8%)	1387 (6.0%)
Cancer Related Pain	751 (1.2%)	348 (1.0%)	345 (1.5%)
Epilepsy	388 (0.6%)	189 (0.6%)	185 (0.8%)
Total Reported Conditions			
Chronic Pain	35,016 (57.0%)	19,920 (59.2%)	13,233 (57.0%)
Anxiety	25,639 (41.8%)	12,255 (36.4%)	10,689 (46.1%)
Back & Neck Problems	18,519 (30.2%)	5638 (29.9%)	4858 (29.2%)
Insomnia	16,343 (26.6%)	10,045 (24.7%)	6783 (27.4%)
Depression	14,662 (23.9%)	5254 (19.1%)	4841 (28.4%)
Post Traumatic Stress Disorder	11,298 (18.4%)	8295 (16.8%)	6362 (20.9%)
Arthritis	10,995 (17.9%)	1130 (15.6%)	1067 (20.9%)
Headaches	9395 (15.3%)	6434 (12.6%)	6600 (18.7%)
Muscle Spasms	8870 (14.5%)	2772 (14.0%)	3932 (15.7%)
Migraines	7308 (11.9%)	4718 (8.2%)	3641 (16.9%)
ADD/ADHD	5614 (9.1%)	3037 (9.0%)	1847 (8.0%)
Chronic Nausea	5366 (8.7%)	2369 (7.0%)	2687 (11.6%)
Fibromyalgia	2535 (4.1%)	347 (1.0%)	1984 (8.5%)
Cancer Related Pain	2392 (3.9%)	4245 (3.4%)	4336 (4.6%)
Epilepsy	1014 (1.7%)	561 (1.7%)	409 (1.8%)

Table 7 reports the top 15 comorbid and total conditions by the total sample and by gender. Patients could report multiple comorbid conditions for which they were seeking medical cannabis. Total reported conditions summarizes the combined primary conditions and comorbid conditions. Patients could not report the same condition for both their primary condition and a comorbid condition. Percentages are calculated from the respective sample size for each column. *ADD* attention deficit disorder, *ADHD* attention deficit hyperactivity disorder

Taking into consideration all medical conditions reported (both primary and comorbid (Table 7)), over half of the sample reported unspecified chronic pain (57.0%), followed by anxiety (41.8%), back and neck problems (30.2%), insomnia (26.6%) and depression (23.9%). Patients reported an average of 3.7 total medical conditions (SD = 2.6). Younger age and female gender were significant predictors of the number of conditions patients reported (*p* = 0.003 and *p* < 0.001, respectively). Females reported an average of 4.1 (SD = 2.9) total conditions, compared to an average of 3.3 (SD = 2.3) among males.

## Discussion

We conducted an extensive retrospective study with the objective of describing the population of patients seeking treatment with medical cannabis at 33 clinics in the US. Our results indicate that patients seeking medical cannabis in the US most commonly report suffering from unspecified chronic pain (57.0%), regardless of age or gender, which is consistent with similar studies that report 61.2 to 82.6% of patients seeking medical cannabis for chronic pain (Boehnke et al. 2019; Sexton et al. 2016; Eurich et al. 2019; Reinarman et al. 2011). Second to unspecified chronic pain, patients were most likely to report anxiety as their primary medical condition, and anxiety was the most commonly reported comorbid condition. This finding is consistent with results from a survey completed by Sexton et al. among self-identifying medical cannabis patients, in which the second and third most common medical conditions that patients reported using medical cannabis for were anxiety (58.1%) and depression (50.3%) (Sexton et al. 2016). Gender was a significant predictor for most primary conditions, which is unsurprising as males and females have different risk factors, experiences and perceptions of illness and do not tend to report or be diagnosed with medical conditions in equal proportions (Seeman 1997; Buvinić et al. 2006; Westergaard et al. 2019).

The average number of conditions and comorbidities is not commonly stated, but has been reported at 1.8 and 3.0 in similar studies, both lower than our findings (Reinarman et al. 2011; Salazar et al. 2019). The average number of conditions reported differed between males and females, with females reporting a higher average number of conditions. This aligns with previous research that has demonstrated that females access health care services more than males and may be more diligent with providing relevant information, which may partially explain why females tend to have higher reported rates of morbidity (Bertakis et al. 2000; Verbrugge and Wingard 1987; Waldron 1983; MacIntyre et al. 1999).

Similar to the survey by Salazar et al., this study also demonstrated the wide variety of conditions for which patients access medical cannabis (Salazar et al. 2019). Conditions representing less than 1.0% of sample accounted for 10.6% of primary conditions reported and included more than 200 unique conditions (Table 8), the majority of which came from states where physicians are able to use their discretion for patients’ qualification (MA, MD, ME, MO). The information on the number and variety of conditions for which patients report seeking medical cannabis treatment is important for medical practitioners for several reasons. Firstly, it highlights the breadth of conditions for which patients are seeking medical cannabis for symptomatic relief. This is important as it identifies patients who may potentially turn to them with questions regarding their suitability for medical cannabis or who may already be seeking medical cannabis without their knowledge, demonstrating the need for practitioners to educate themselves and be prepared to discuss and provide their professional medical opinion. Secondly, these data demonstrate that patients seeking medical cannabis are complex patients who have more than a single ailment. While rigorous clinical trials are still needed to validate the use of medical cannabis for these conditions, real world data are also needed to describe these patients, as complex patients are more likely to be excluded from clinical trials evaluating the effectiveness of a medication (Hanlon et al. 2019). Finally, these data highlight the potential utility of medical cannabis and how it is currently utilized for treatment of multiple conditions with which a patient is suffering.

**Table 8:** Summary of “other” conditions each representing less than 1.0% of the total sample of patients seeking medical cannabis certification

Condition	Patients, n (%)
	***n*** = 6519
Neuropathic Pain	580 (0.9%)
Spinal Cord Injury/Disease	574 (0.9%)
Glaucoma	480 (0.8%)
Crohn’s Disease	472 (0.8%)
Stress	418 (0.7%)
Multiple Sclerosis	396 (0.6%)
Irritable Bowel Syndrome	364 (0.6%)
Mood Disorders	271 (0.4%)
Acute Pain	253 (0.4%)
Colitis	244 (0.4%)
HIV/AIDS	224 (0.4%)
Opiate Dependence	183 (0.3%)
Scoliosis	156 (0.3%)
Appetite Stimulation	152 (0.2%)
Parkinson’s Disease Symptoms	148 (0.2%)
Hepatitis C	133 (0.2%)
Chemotherapy Induced Nausea	108 (0.2%)
Autism	57 (0.1%)
Cachexia/Wasting Syndrome	56 (0.1%)
Movement Disorder	46 (0.1%)
Anorexia	42 (0.1%)
Obsessive Compulsive Behaviour	41 (0.1%)
Tremors	38 (0.1%)
Alzheimer’s Disease	26 (0.0%)
Bipolar Disorder	25 (0.0%)
Tourette’s Syndrome	10 (0.0%)
Other	1022 (2.0%)

Table 8 reports the “other” conditions reported in Table 5 that each represent less than 1.0% of the total sample. Percentages are given out of the total sample (61,379 patients). There are over 200 unique conditions that were manually entered into the database by clinic staff that are categorized as “Other” in this Table. *HIV* human immunodeficiency viruses, *AIDS* acquired immunodeficiency syndrome

Medication use and average number of medications increased with age; however, patients reported medication use less than the general US population overall. Findings from the National Health and Nutrition Examination Survey reported that 83.6% of adults aged 60 and over used prescription medication in the previous 30 days, compared to only 56.4% of our sample aged 60 and older. For adults aged 40–59, our sample also reported less medication use; 48.3% compared to 59.5% (Martin et al. 2015). This difference may be a result of patients under-reporting their medications at the clinic, or medication information not being correctly transcribed into the software from practitioners’ notes. Alternatively, it may show a true difference in characteristics between the general US population and those accessing medical cannabis. The latter may suggest that those accessing medical cannabis may be doing so in lieu of using traditional pharmaceutical medications; however, this theory contradicts results of a study from 2018 that reported that medical cannabis users are more likely to use prescription medications (Caputi and Humphreys 2018). Reported medication use was not analyzed in reference to specific primary medical conditions for the purposes of this study, but identified it as an area of interest for future research. It is interesting to note that 26.1% of the sample did not report using medications or alternate therapies at the time of their initial assessment. This may demonstrate a limitation of the data, as there is potential that there is under-reporting at the system level; however, this may also indicate that patients are seeking medical cannabis where other treatments have failed, which would benefit from further investigation.

When comparing our results to a study of 1746 patients attending assessment clinics in California in 2006, who were primarily male (72.9%) and between the ages of 25–54 (69.2%) (Reinarman et al. 2011), it suggests that the medical cannabis patient population has evolved over time to include more females**,** and a wider range of ages; 40% of the sample who reported gender were female, and 31.5% of the patient population in this study were over the age of 54. The increasing age of medical cannabis users and increase in female users were also reported in a review completed by Fairman et al. (Fairman 2016). The high representation of Caucasians in this sample is consistent with the literature in which Caucasians represent the majority of medical cannabis users reported in other studies (77.0, 86.5, 61.5%) (Sexton et al. 2016; Reinarman et al. 2011; Reiman 2007). This is substantiated by the fact that Caucasians are the racial majority in the US, particularly in the states where the clinics are located.

The median estimated household income in the ZIP codes where our patient sample resides was higher than the US median, $69,481 compared to $61,937 (Bureau UC 2019). When taking into consideration the median household income from just the included states, $58,912 (US Income Statistics - Current Census Data for Zip Codes 2018), the median estimated household income from our sample was still higher; however, as the income data for our sample was a surrogate, this may be inflated and potentially inaccurate. Similar studies tend to report that income among medical cannabis users is lower than the average, which highlights the need for additional investigation (Sexton et al. 2016; Reiman 2007).

Data collected as part of the intake for patients in this retrospective study provide an interesting perspective on cannabis experience prior to seeking medical certification, as almost 70% reported using cannabis prior to their certification. This number is substantially higher than the lifetime cannabis use estimate among Americans from the National Survey on Drug Use and Health, which reported a lifetime cannabis use of 45.3% in 2018 (2018 NSDUH Detailed Tables | CBHSQ Data 2019). Unfortunately, it is not known how many of the patients in the dataset had medical certification from a separate clinic or physician elsewhere prior to becoming a patient at a CB2 Insights clinic.

Strengths of this study include the large sample of patients accessing medical cannabis across 12 states. The consistent input of data at the clinics allowed for a comprehensive review of characteristics, and most importantly, provided data on all medical conditions for which patients sought medical cannabis, rather than just one per patient. Additionally, data collection was verified by medical professionals at the time of input. Limitations of this study primarily include missing data (i.e., a large number of patients for whom gender and race were not reported), a lack of ethnicity data, the absence of data on patients who did not qualify for medical cannabis certification and were not included in the registry, and the use of surrogate income data. Another limitation is that the data came from a single network of clinics, and do not represent patients in all states where medical cannabis is legal.

## Conclusion and future initiatives

This retrospective study offers insight into the characteristics and commonly reported type and number of medical conditions among patients accessing medical cannabis in the US. It highlighted the conditions that patients are seeking medical cannabis for most often that would benefit from further clinical evidence; mainly pain conditions, anxiety, insomnia, depression and PTSD. This study also demonstrated that patients often use medical cannabis to treat more than one condition, which is important for the medical community to understand and be aware of, as well as the patients who may be turning to cannabis as a treatment option. This finding in particular raises questions that are important to investigate, including why patients use medical cannabis for multiple conditions and whether they use different products to treat their various symptoms. As this study explored demographic and medical characteristics from patients in 12 different states, an in-depth review comparing states with contrasting cannabis regulations would offer further insights into medical cannabis use and access in the US.

## Data Availability

The datasets used for this article are not publicly available due to patients’ privacy. Data can be made available upon appropriate request to the authors.

## Abbreviations

CBD: cannabidiol
CI: confidence interval
IQR: interquartile range
IRB: institutional review board
PTSD: post-traumatic stress disorder
SD: standard deviation
THC: Δ-9-tetrahydrocannabinol
US: United States
